# Can Alzheimer's Disease Be Secondary to Type-2 Diabetes Mellitus?

**DOI:** 10.7759/cureus.31273

**Published:** 2022-11-08

**Authors:** Vani Jangra, Jeshnu Tople

**Affiliations:** 1 Medicine and Surgery, Jawaharlal Nehru Medical College, Datta Meghe Institute of Medical Sciences, Wardha, IND; 2 Anaesthesiology, Jawaharlal Nehru Medical College, Datta Meghe Institute of Medical Sciences, Wardha, IND

**Keywords:** obesity and diabetes, early-onset dementia, insulin resistance, alzheimer’s dementia, diabetes mellitus type 2

## Abstract

Alzheimer's disease and insulin resistance are prevalent in older adults. Insulin's ability to effectively affect target tissues is diminished by IR. Hyperglycemia, higher blood pressure, elevated triglyceride levels, decreased HDL levels and central obesity are the outcomes of a condition, namely metabolic syndrome. Cognitive impairment and abnormalities of the brain have been linked to metabolic syndrome (MetS), a grouping of risk factors for type 2 diabetes mellitus. Type-2 diabetes mellitus and its relationship to other conditions have been investigated on the assorted extent in the pair of, human and animal subjects. First, it was shown that insulin receptors are present in the brain, namely the hippocampus. Most insulin is delivered to the brain by crossing the blood-brain barrier. Second, numerous research revealed that insulin impacts various neurotransmitters in a way that enhances memory and cognition. Thirdly, several pathological research has also shown that beta-amyloid plaques, hyperphosphorylated tau protein, and brain shrinkage, particularly in the hippocampus, are shared brain lesions between insulin and Alzheimer's disease. In light of this, type 2 diabetes mellitus may be viewed as a liability for dementia and Alzheimer's disease.

## Introduction and background

The characteristic of diabetes mellitus (DM) is hyperglycemia brought on by a deficiency in insulin, an inability to utilize insulin, or both. It is associated with the origination of secondary problems that lead to several comorbidities. The correlation between DM and Alzheimer's disease (AD) is becoming more and more apparent. A hallmark and indication of AD pathology is increased tau protein phosphorylation (hyperphosphorylation), which is caused by a buildup of neurofibrillary tangles, which in turn is brought on by impaired insulin signaling in the brain. Glycogen synthase kinase-3 (GSK-3) signaling has been connected to the pathogenic and physiological processes in DM and AD, respectively. This may explain why DM patients have a higher chance of getting AD as they age and their disease progresses [1]. DM is considered an independent risk factor for cognitive impairment or dementia, particularly in cases when AD is present. Insulin insufficiency resulting from pancreatic islet beta cell death (often autoimmune) underlies type 1 DM (T1DM) [2]. The most prevalent kind of diabetes, type 2 DM (T2DM), is brought on by insulin resistance in peripheral tissues and is most usually linked to aging, having a family history of the disease, being overweight, and not exercising. Regardless of subtype, etiology, pathophysiology, or insulin availability, DM syndromes are characterized by deficiencies in insulin actions and signaling that cause chronic hyperglycemia. These abnormal neural alterations cause neuronal death and cognitive impairment as AD progresses. Clinically, patients with AD experience a gradual loss of memory and a deterioration in cognitive abilities. These include challenges with memory, language, problem-solving, and other mental skills that weaken a person's capacity to carry out daily tasks [3]. Some experts have referred to AD as type 3 DM due to the higher incidence of late-onset AD in DM [4]. This review will analyze the connections between T2DM and dementia at many levels.

## Review

Potential contributions of obesity and T2DM to the pathogenesis of AD

Prevalence rates for diabetes, obesity, and AD have been seen to increase any form of heart problem, which results in decreased cerebral blood flow (CBF). This impairs the vascular homeostasis of the brain and exacerbates any cognitive issues brought on by the accumulation of tau and amyloid proteins [5]. Obesity has been considered an independent risk factor for dementia and AD, in addition to being a known risk factor for T2DM, cardiovascular disease, and cancer [6]. Epidemiological research supports a significant correlation between T2DM and dementia and suggests that T2DM is a considerable risk factor for AD [7]. Adipose tissue produces the peptide hormone leptin, which primarily controls food intake. It has been demonstrated that insulin stimulates leptin production to promote satiety when adipocytes are exposed to glucose. In contrast, by a negative feedback mechanism, leptin reduces insulin release and increases tissue sensitivity, resulting in glucose uptake for energy use or storage. Leptin resistance and insulin resistance are thus strongly connected. Middle-aged obesity and AD have been linked [8]. Leptin is mainly attached to its soluble receptor in lean participants, in contrast to obese people with a higher percentage of the hormone in the free form. Plasma leptin levels decrease when adipose tissue shrinks, and when it grows, leptin levels rise, and appetite is suppressed [9]. All these actions are mediated by binding to specific leptin receptors (LepR), which are present in both peripheral tissues and the central nervous system (CNS). The arcuate, ventromedial, paraventricular, and ventral premammillary nuclei of the hypothalamus are where LepR is found. However, LepRs are also found in other regions that are mostly unrelated to energy balancing, including the neocortex, hippocampus, thalamus, leptomeninges, and choroid plexus [10]. The role of insulin and leptin hormones in memory and cognitive function, which are conventionally linked to diabetes and obesity, is attracting more and more attention. Impairment in brain functioning can be correlated with digressive signaling by these hormones [11]. 

Common risk factors for DM and AD

Diabetes patients who experience cognitive abnormalities usually struggle in the following areas: executive function, learning and memory, attention, learning and motor efficiency, and psychomotor efficiency. After adjusting for diabetic vascular disease and insufficient cerebral circulation, there has been increased cortical and subcortical shrinkage. The molecular, biochemical, and histological abnormalities in AD may be caused by disturbances in brain insulin signaling pathways [12].

Due to the anticipated rise in age-related illnesses, it is vital to understand how DM raises the risk of AD and the underlying mechanisms. Some intriguing similarities between diabetes and AD are: The beta-amyloid protein, a defining feature of AD, was discovered to rise dramatically in persons with high blood sugar levels, including those with T2DM. Deteriorations in cognitive function and modifications in brain structure are linked to DM [13]. Participants in one study displayed elevated levels of insulin resistance in the brain and diminished capacity to utilize glucose as fuel for essential brain function [14]. Table 1 summarizes the association between AD and T2DM.

**Table 1:** Collocation between Alzheimer's disease and type 2 diabetes mellitus

Common risk factors between Alzheimer’s disease and type 2 diabetes mellitus	Common occurrence of beta-amyloid and islet amyloid polypeptide
Cerebral vascular disorders	Amyloidogenic proteins
Genetic variations	Membrane disruption
Dyslipidemia	Oxidative damage
Obesity	Inflammation
Amyloid plaque deposition	Mitochondrial dysfunction
Insulin resistance	Apoptosis

A framework that mechanistically connects all these events will be necessary for a deeper understanding of the pathophysiology of Alzheimer's disease. Even though the additional knowledge is inconsistent or riddled with contradicting and unresolved ideas regarding the possible distributors of T2DM, the metabolic syndrome, and obesity to AD etiology, insulin insufficiency, and insulin resistance as mediators of AD-related neurodegeneration [15].

Risk factors and neuropathology usually include the medial temporal lobe, the area of the cerebral cortex most severely impacted in AD, followed by the atrophy of neurons. The forebrain and hippocampal regions are where pathology seems to begin, spreading later to the frontotemporal cortices. It generally bypasses the cerebellum as it extends to the striatum and thalamus. A variety of 36-43 residue-long amyloid peptides, which go through the formation of fibrils to create alpha sheets that are resistant to breakdown, make up the majority of senile plaques. The hippocampus formation, frontal region, and neocortical regions are all affected, and subsequently, the striatum and thalamus, after spreading farther over the cerebral cortex. Neurofibrillary tangles are only discovered in areas where amyloid was already present, indicating that the pathology of amyloid appears to precede that of tau.

Comorbidities as Risk Factors

Vascular Diseases: They have a significant role played by the neurovascular unit, a specialized grouping of neurons, astrocytes, and vascular endothelial cells. Changes in this vascular system result in decreased global cerebral perfusion, which impairs cognition and causes brain dysfunction, thereby initiating the concept of AD. The primary risk factor for stroke, a condition that deprives the brain of blood flow, is hypertension. In reality, diabetes patients experience more severe strokes, which raises the mortality rate. Other cardiovascular disorders in the aging and AD brain have been described, providing evidence that cerebrovascular dysfunction lowers the threshold for acquiring AD dementia and contributes to neurodegeneration and cognitive failure [16].

Amyloid-Forming Diseases: The presence of insoluble protein aggregates with a fibrillary form in the brain and pancreas, respectively, is a hallmark of the amyloid-forming illnesses AD and T2DM. Most patients have proteinaceous plaques, which are predominantly made of islet amyloid polypeptide (IAPP), which is one of the main secretory products of the pancreatic β-cells [17]. Evidence suggests that the presence of an amyloid precursor protein sequence in the IAPP molecule could increase the IAPP secretion from β-cells associated with the elevated insulin demand and abnormalities in the processing of pro-IAPP contribute to aggregation in T2DM, causing cellular dysfunction and subsequent membrane disruption, channel formation, and toxicity. However, the exact mechanism by which normally soluble IAPP can form toxic aggregates is unknown [18].

Hyperglycemia: This is one of the critical features of insulin resistance, metabolic syndrome, and diabetes and can be brought on by long-term exposure to a diabetic environment, high-fat/high-sugar meals, and physical/mental stress. As a result, diabetic neuropathy, nephropathy, artery damage, and cardiovascular illnesses are among the pathological problems brought on by the resulting glucotoxicity that damages peripheral tissues and vessels. Considering how closely hyperglycemia is linked to the onset of dementia and cognitive impairment, there may be a causal link between the two conditions.

Diabetes and dementia have complicated etiologies. There is some overlap in the risk factors for these two disorders. Inflammation, oxidative stress, and mitochondrial dysfunction are a few shared pathways between diabetes and dementia. According to theories, glucotoxicity may cause morphological and functional impairment in brain and nerve cells, hemorrhage in the cerebral blood vessels, and an elevated buildup of amyloid beta [19]. It is well established that amyloid beta accumulation in brain lesions, and brain insulin resistance is made worse by neuroinflammation, oxidative stress, and mitochondrial dysfunction. Upon interacting with pattern recognition receptors on microglia and astrocytes, misfolded and clumped proteins trigger an innate immune response that releases inflammatory mediators and worsens the condition [20].

The impact of diabetes medicines on cognitive function

**Figure 1:**
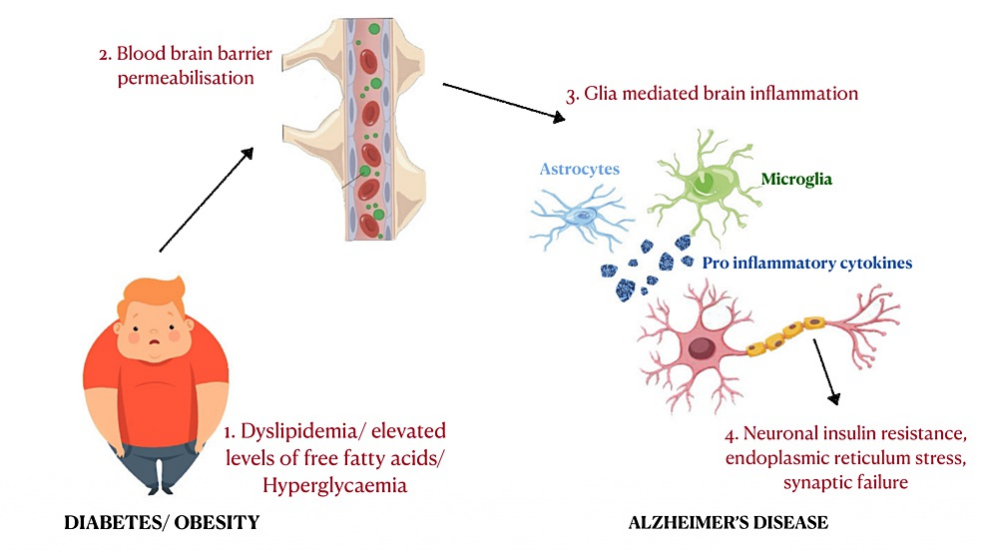
Insulin resistance interaction in Alzheimer's disease and type 2 diabetes mellitus Figure credit: Vani Jangra

The effects of diabetic drugs on cognitive function along with drugs that are designed to raise glucose levels near the non-diabetic range and their associated increased risk of hyperglycemia have presented with raised concerns about the effects of diabetes mellitus on cognitive performance increased over time [21]. Metformin improves tissue insulin sensitivity by decreasing hepatic gluconeogenesis. It quickly penetrates the blood-brain barrier [22]. Moreover, via enhancing mitochondrial function, it appears to have a neuroprotective and anti-neuronal aging impact [23]. Human-based observational studies have demonstrated that diabetic individuals on metformin have reduced mild cognitive impairment and improved symptoms of dementia when compared to those taking other antidiabetics or no medication at all [24]. By inhibiting potassium channels on beta cells, sulfonylureas promote insulin secretion. However, glimepiride was discovered to lessen amyloid's impact on synaptic degeneration in a laboratory setting [25]. Incretins called GLP-1 receptor agonists, which get released by the intestines, inhibit stomach motility, boost insulin production, and suppress glucagon production. Blood-brain barrier-crossing GLP-1 receptor antagonists have been identified, and the brain, particularly the hippocampus, contains GLP-1 receptor antagonists [26]. Due to the saturable passage across the blood-brain barrier, insulin does concentrate in the cerebrospinal fluid when administered parenterally. Attempting to raise the dose is constrained by the danger of hypoglycemia. However, it can cross the blood-brain barrier when administered intranasally, allowing us to reach a significant amount of it in the brain [27]. According to in vivo research, insulin aids in the elimination of beta-amyloid, metabolism of the beta-amyloid precursor protein, and modulation of tau protein phosphorylation [28]. Additionally, intranasal insulin improves memory performance in those with advanced AD, mild cognitive impairment, and healthy people, which also retains the volume of the brain regions influenced by AD [29]. Figure 1 demonstrates how insulin resistance and comorbidities like obesity, can be a link between T2DM and AD.

What is affected by the brain's insulin levels?

Chronic peripheral insulin increases and decreased brain insulin levels and activity are the hallmarks of insulin resistance. The effects of peripheral hyperinsulinemia on memory, CNS inflammation, and control of the amyloid peptide are among the potential ways these risks are raised [30]. T2DM is characterized by insulin resistance, also becoming known as a possible characteristic of AD and related dementia [31]. However, it is still unknown what causes cognitive deterioration and changes in brain structure in obesity and T2DM. Given that acute and chronic blood glucose dysregulation has been associated with impaired cognition abilities [32], most research on pre-diabetes and T2DM has concentrated on how glycemia extremes affect people. Insulin signaling within the brain has recently drawn increasing attention due to its significance in brain function. This is due, in part, to the fact that significant changes in brain insulin action have been linked to dementia and brain aging in addition to obesity and T2DM. Therefore, it has been suggested that a possible connection between metabolic and cognitive dysfunctions is caused by reductions in the sensitivity of central neuronal pathways to insulin, also known as brain insulin resistance [33,34]. The binding of insulin activates specialized transporter proteins to dimerized receptors, facilitating the influx of glucose. However, neurons have additional ways to get their hands on glucose, such as transporters that are not insulin-dependent [35]. When a person is obese, has visceral fat buildup, or has a specific genetic background, their brain can become insulin resistant. A potential strategy for preventing and treating metabolic illnesses like T2DM may involve overcoming brain insulin resistance [36]. 

ApoE-ε4

Early-onset Alzheimer's disease is more likely when ApoE-4 is expressed, which is also linked to diabetes. It can deposit more of the neurotoxic compound A and hinder its removal [37]. In addition to altering the cholesterol transporter protein ABCA, ApoE-4 is less protective against oxidative stress and causes cholinergic dysfunction in AD.

## Conclusions

DM and AD are becoming more prevalent among the elderly. The same pathogenic causes can cause both illnesses. In this review, we broadened the understanding of fundamental molecular pathways that connect these two diseases and aimed to emphasize the growing body of evidence that demonstrates the same pathophysiology of T2DM and AD. More profound knowledge of their connection might help to improve the control of both diseases. More studies are required to fully comprehend the effects of mild to moderate declines in cognitive function on diabetic individuals. DM is usually detected at an older age and is frequently linked to obesity, insulin resistance, hypertension, and dyslipidemia, all of which can have a detrimental effect on the brain. Considering the importance of nerve damage in AD, we can test more for nerve endings and skin biopsies for immunological findings presumably related to T2DM. For cytological studies, biomarkers can be tested. Cross-sectional studies among the population can be helpful for a better understanding of the underlying pathophysiology of AD and T2DM. 
